# Social determinants and spatio-temporal variation of Ischemic Heart Disease in Manitoba

**DOI:** 10.1186/s12889-021-12369-1

**Published:** 2021-12-30

**Authors:** Justin Dyck, Robert Tate, Julia Uhanova, Mahmoud Torabi

**Affiliations:** 1 Department of Community Health Sciences, University of Manitoba, Winnipeg, Canada; 2 Department of Internal Medicine, University of Manitoba, Winnipeg, Canada

## Abstract

**Introduction:**

The aim was to study any spatial and/or temporal patterns of ischemic heart disease (IHD) prevalence and measure the effects of selected social determinants on these spatial and space-time patterns.

**Methods:**

Data were obtained from the Population Research Data Repository housed at the Manitoba Centre for Health Policy to identify persons who were diagnosed with IHD between 1995 and 2018. These persons were geocoded to 96 geographic regions of Manitoba. An area-level socioeconomic factor index (SEFI-2) and the proportion of the population who was Indigenous were calculated for each geographic region using the 2016 Canadian Census data. Associations between these factors and IHD prevalence were measured using Bayesian spatial Poisson regression models. Temporal trends and spatio-temporal trends were measured using Bayesian spatio-temporal Poisson regression models.

**Results:**

Univariable models showed a significant association with increased regional Indigenous population proportion associated with a higher prevalence of IHD (RR: 0.07, 95% CredInt: (0.05, 0.10)) and for SEFI-2 (RR: 0.17, 95% CredInt: (0.11, 0.23)). Using a multivariable model, after accounting for the proportion of the population that was Indigenous, there was no evidence of an association between IHD prevalence and area-level socioeconomic factor. Spatio-temporal models showed no significant overall temporal trend in IHD prevalence, but there were significant spatially varying temporal trends within the 96 regions.

**Conclusions:**

Association between Indigenous population proportion and IHD is consistent with previous research. No significant overall temporal trend was measured. However, regions with significantly increasing trends and significantly decreasing trends in IHD prevalence were identified.

**Supplementary Information:**

The online version contains supplementary material available at 10.1186/s12889-021-12369-1.

## Background

Ischemic Heart Disease (IHD) is a chronic disease that occurs due to a narrowing of arteries resulting in reduced blood flow to heart tissue. This disease has high prevalence rates in both Manitoba and Canada as a whole, which have been measured as 8.5% of all Canadians aged 20+ and 8.3% of all Manitobans aged 19+ [1, 2].

Manitoba is a sparsely populated Province in central Canada, with the majority of its population living in the main city centre of Winnipeg. Manitobans from different backgrounds and economic standings are affected by IHD, however, it has been shown that it does not affect people of different demographic and/or socioeconomic status (SES) stratums equally [3, 4]. Thus, it should be expected that some of the geographical variation in disease prevalence could be attributed to the geographical distribution of demographic and SES factors. This is compounded by the natural phenomena that nearer regions are in general more highly correlated than further regions [5]. The aim of this study was to study spatial and temporal patterns of IHD prevalence in Manitoba over a 20-year period using both spatial and spatio-temporal models, while also measuring the effects of selected social determinants on these spatial and space-time patterns. As such, we predicted that positive relationships exist between IHD prevalence and lower SES as well as increased Indigenous population proportion within spatial areas after accounting for inherent spatial and spatio-temporal confounding effects. For this study, Indigenous is taken to mean anyone self-identifying as First Nations, Inuit, or Metis.The general provincial trend in Manitoba has been a decline in both IHD prevalence and incidence over time, which is an indication that the burden of disease has lessened over recent years, specifically the 2002-2007 to 2007-2012 time periods [1]. This is consistent at the national level, with decreasing trends in IHD prevalence and incidence for recent times in Canada [6]. However, some of the finer details get lost when inferring about this disease at these large geographic aggregation schemes, where these trends may not be consistent across all age-groups, ethnicities, or finer geographically aggregated regions. IHD prevalence and incidence rates have been shown to be rising significantly over these time periods in some health districts in Manitoba, such as the Cross Lake and Norway House districts, contrary to the provincial trend [1]. Understanding how a leading cause of premature deaths in Manitoba is affecting some geographic regions differently from others is important as equitable healthcare for all Manitobans is a priority for Manitoba’s health care system.

Some of the main behavioural risk factors of IHD, such as alcohol consumption, sedentary lifestyles, and smoking habits, [7] have been shown to be more prevalent in environments where more economic deprivation exists [8–10].  Economic deprivation has also been shown to occur in regions with higher environmental deprivation, such as higher levels of pollution, further illustrating a non-equal distribution of IHD risk across geographical space [11, 12].

Demographic structures have also been shown to be associated with changes in IHD risk, with Indigenous persons having different rates of cardiovascular disease than non-Indigenous persons [3]. Tobe et al. (2018) showed that cardiovascular disease among Indigenous populations is not decreasing at the same rate as in the rest of the population. While prevalence rates of cardiovascular disease among Indigenous men decreased more slowly than among the non-Indigenous population, the prevalence rates among Indigenous women have increased [3]. This is likely linked to findings where Indigenous Canadians living off-reserve were measured to have higher rates of tobacco smoking, second-hand smoke exposure, and alcohol consumption than non-Indigenous Canadians [13]. Other important factors such as food insecurity and working poverty status were found to be significantly higher in Indigenous populations, leading to higher rates of economic and environmental deprivation [13]. Manitoba has the highest proportion of Indigenous population out of all Canadian provinces at approximately 18%, and is thus an important variable to consider in analyzing IHD in Manitoba. Here, the geographic distribution of Indigenous populations within Manitoba is highly variable with high proportions in the northern regions and low in the south (Statistics Canada, 2016) [14] motivating the need to study this factor for IHD at the ecological level.

## Methods

### Data Sources and Study Measures

This study was a population-based ecological study using administrative health records housed at the Manitoba Centre for Health Policy (MCHP), with a linkage to Statistics Canada’s 2016 Census records. MCHP’s Population Research Data Repository (PRDR) included four datasets of interest: the Manitoba health insurance registry, medical/physician claims, hospital abstracts (outpatient records), and the drug program information network (DPIN) datasets. These datasets were then linked at the individual level using the scrambled personal health identification numbers (PHINs).

This study’s time-period was from the 1995-1996 fiscal year to the 2017-2018 fiscal year (April 1, 1995 – March 31, 2018), which was chosen due to the availability of the data in the PRDR, and the study cohort was comprised of all Manitobans that have had active Manitoba Health Insurance at some point within this period and was restricted to individuals between the ages of 40 and 85. IHD prevalence was calculated by identifying individuals that have had at least one instance of IHD in the hospital abstracts or at least two instances in the medical claims dataset, or a combination of at least one instance in the medical claims dataset and two prescriptions in the DPIN dataset within a five year period. This inclusion algorithm was designed to ensure an adequate level of specificity for IHD prevalence [15, 16]. After these persons were identified as having IHD they were counted in the numerator for prevalence until censored for either death, emigration from Manitoba, or aging out of the study.

Geographic classification of individuals was done with the 6-digit postal code and municipality code as defined by Manitoba Health, Seniors & Active Living. Individuals were aggregated into the 71 regional health authority districts (RHAD) for regions outside Winnipeg, and the 25 neighbourhood clusters for within Winnipeg regions using the algorithm available from MCHP and Manitoba Health for assigning Health Districts and Neighbourhood Clusters from municipality and postal codes. Although some District boundaries have changed over the study period, we used the current boundaries throughout the study period to ensure comparable results. Some geographic misalignment does occur in a small number of cases between postal code and District boundaries, where in this case the District with the higher proportion of area of the postal code is used. For the remainder of the paper, the term RHAD is used to define all 96 areal units (geographic regions).

Indigenous population proportion was calculated at the RHAD level using the 2016 Census and is assumed to be stationary over the study period. A SES measure was constructed at the RHAD level from the 2016 Census based on the socio-economic factor index (SEFI) which was defined by researchers at MCHP. This index was designed to bridge socio-economic factors that are highly influential on potential years of life lost, premature mortality rates, life expectancy, and self-rated health [17]. SEFI-2 is the second iteration of this algorithm and is the definition used for this study as a proxy for SES. This is defined as the scores of a factor analysis of the four 2016 Census variables of median household income, proportion of high school graduates, unemployment rate, and proportion of single-parent families [17].

### Statistical Analysis

The outcome for subsequent statistical models was expressed as a standardized risk ratio. Standardization was done to factor out the effects of age and sex in the analyses, as it is well known that these contribute to IHD risk [18–20]. This was done via indirect standardization to the Provincial population using 5-year age groups.

To examine the contribution of the covariates, a mixed Poisson model (MPM) was used to assess each covariate alone, as well as together for any potential confounding. The spatial random effects terms in these models were defined using the Besag-York-Mollie (BYM) specification [21]. This modification of the Poisson regression model includes two additive random effects terms; one spatially correlated heterogeneity term, and the other a non-correlated heterogeneity term which were used to account for any spatially structured covariance between the RHADs. Since spatial covariance was present, as indicated by a significant Moran’s Index test, which violated the assumption of independent and identically distributed residuals in the regular Poisson regression model, this modification was needed to satisfy these assumptions. The data used for this model included the 5-year time frame of 2013-2017 calendar years inclusive, due to this being the most recent 5-year period available at the time of analysis. The model equation for this model is given as in (1).

$${\mathrm Y}_{\mathrm i}\sim\mathrm{Poisson}\left({\mathrm\lambda}_{\mathrm i}\right)$$

1 $$\log\left({\mathrm\lambda}_{\mathrm i}\right)=\log\left({\mathrm E}_{\mathrm i}\right)+{\mathrm X}_{\mathrm i}\mathrm\beta+{\mathrm v}_{\mathrm i}+{\mathrm u}_{\mathrm i}$$

where Y_i_ is the number of IHD prevalent persons in region i, E_i_ is the expected number of IHD prevalent persons for region i, X_i_ is the covariate vector for region i, v_i_ is the spatially unstructured term, and u_i_ is the spatially structured term for region i. Indirect age-sex standardization was done through the E_i_ term, which is defined as:

$${E}_{i}= {\sum }_{j}\frac{{Y}_{j}^{\left(s\right)}}{{n}_{j}^{\left(s\right)}}{n}_{ij}$$

where Y_j_^(s)^ and n_j_^(s)^ are the disease counts and population totals in the Provincial population for the j^th^ age-sex group, and n_ij_ is the population in the j^th^ age-sex group in the i^th^ RHAD. Age groups were defined using 5-year intervals from 40 to 44 to 80-84. This effectively puts the baseline for the risk ratio’s in the spatial models to be the Provincial prevalence. As described above, the u_i_ and v_i_ are given priors defined by a BYM model, that is:

$$u_i\vert u_j\sim\mathrm{Normal}\left(\frac1{N_i}\sum\nolimits_ja_{ij}u_j,\frac{\sigma_u^2}{N_i}\right)$$

$$v_i\sim Normal\;\left(0,\sigma_v^2\right)$$

where N_i_ is the number of neighbors of region i, and a_ij_ = 1 if the i^th^ and j^th^ regions have common borders. The precisions 1/σ_u_^2^ and 1/σ_v_^2^ were given logGamma(1, 0.0005) priors, where sensitivity analyses were done using flat uniform distributions for σ_u_^2^ and σ_v_^2^ to ensure the priors did not influence subsequent results.

Assessment of the presence of any temporal/spatio-temporal patterns in the IHD data was done using the full cohort of data that was aggregated and broken into yearly segments; 18 yearly segments total. Two temporal random effect terms were included in the model to understand the temporal trends: one unstructured and the other was given a random walk prior. An additional model with a space-time interaction random effect term was also assessed to detect the significance of spatially varying temporal effects. In the latter model, the space-time interaction term’s covariance structure matrix was defined as the Kronecker product of the interacting parameters’ structure matrices, which was used to identify any temporal patterns that vary across the areal units by calculating the portion of model variance attributed to this random effect term compared with other random effects terms. The model equation for the model without the space-time interaction term is given as (2), and with the interaction effect term as (3).

$${\mathrm Y}_{\mathrm{it}}\sim\mathrm{Poisson}\left({\mathrm\lambda}_{\mathrm{it}}\right)$$

2 $$\log\left({\mathrm\lambda}_{\mathrm{it}}\right)=\log\left({\mathrm E}_{\mathrm{it}}\right)+{\mathrm v}_{\mathrm i}+{\mathrm u}_{\mathrm i}+{\mathrm\gamma}_{\mathrm t}+{\mathrm\phi}_{\mathrm t}$$

3 $$\log\left({\mathrm\lambda}_{\mathrm{it}}\right)=\log\left({\mathrm E}_{\mathrm{it}}\right)+{\mathrm v}_{\mathrm i}+{\mathrm u}_{\mathrm i}+{\mathrm\gamma}_{\mathrm t}+{\mathrm\phi}_{\mathrm t}+{\mathrm\delta}_{\mathrm{it}}$$

where Y_it_ is the number of IHD prevalent persons in region i and time t, E_it_ is the expected number of IHD prevalent persons for region i and time t. As in the spatial model, v_i_ is the spatially unstructured term and u_i_ is the spatially structured term using the BYM specification., γ_t_ is the temporally unstructured term, ϕ_t_ is the temporally structured term, and δ_it_ is the space-time interaction term. Indirect age-sex standardization was done in this context through the E_it_ term, which was defined as:

$${E}_{it}= {\sum }_{j}\frac{{Y}_{jt}^{\left(s\right)}}{{n}_{jt}^{\left(s\right)}}{n}_{ijt}$$

where Y_jt_^(s)^ and n_jt_^(s)^ are the disease counts and population of the j^th^ age-sex group for time t for the Provincial population. This puts the baseline for the risk ratio’s for the spatio-temporal models as the Provincial prevalence at time point t.

Bayesian model inference was carried out using the integrated nested Laplace approximation (INLA) approach [22], where this method was chosen due to the complexity of these models. The INLA method is effective if the data can be modelled with a latent Gaussian Markov Random Field (GMRF) [23]. If these conditions are satisfied, the INLA algorithm can be used for Bayesian inference of the model parameters instead of the more popular Markov Chain Monte Carlo (MCMC) sampler, where INLA produces results with much less computation time than the MCMC approach. The Laplace approximation is said to have negligible error when the assumptions are satisfied which were checked via cross-validation. These checks were carried out by assessing the conditional predictive ordinate (CPO) and the probability integral transform (PIT) [23], where all models presented later passed these tests. Priors were selected as uninformatively as possible so as to not bias the results with prior beliefs of the data, and the deviance information criterion (DIC), and the CPO were used to assess model fit.

Analysis was performed in R version 4.0.1 [24], with models being run with the R-INLA package [22], plots generated with the ggplot2 package [25], and map output generated with the tmap package (Tennekes, 2018) [26].

### Ethical Considerations

Prior to data access being granted by MCHP, ethics approval was sought and granted from both the University of Manitoba’s health research ethics board as well as from Manitoba Health, Seniors & Active Living’s health information and privacy committee.

## Results

### IHD Prevalence

**Fig. 1:**
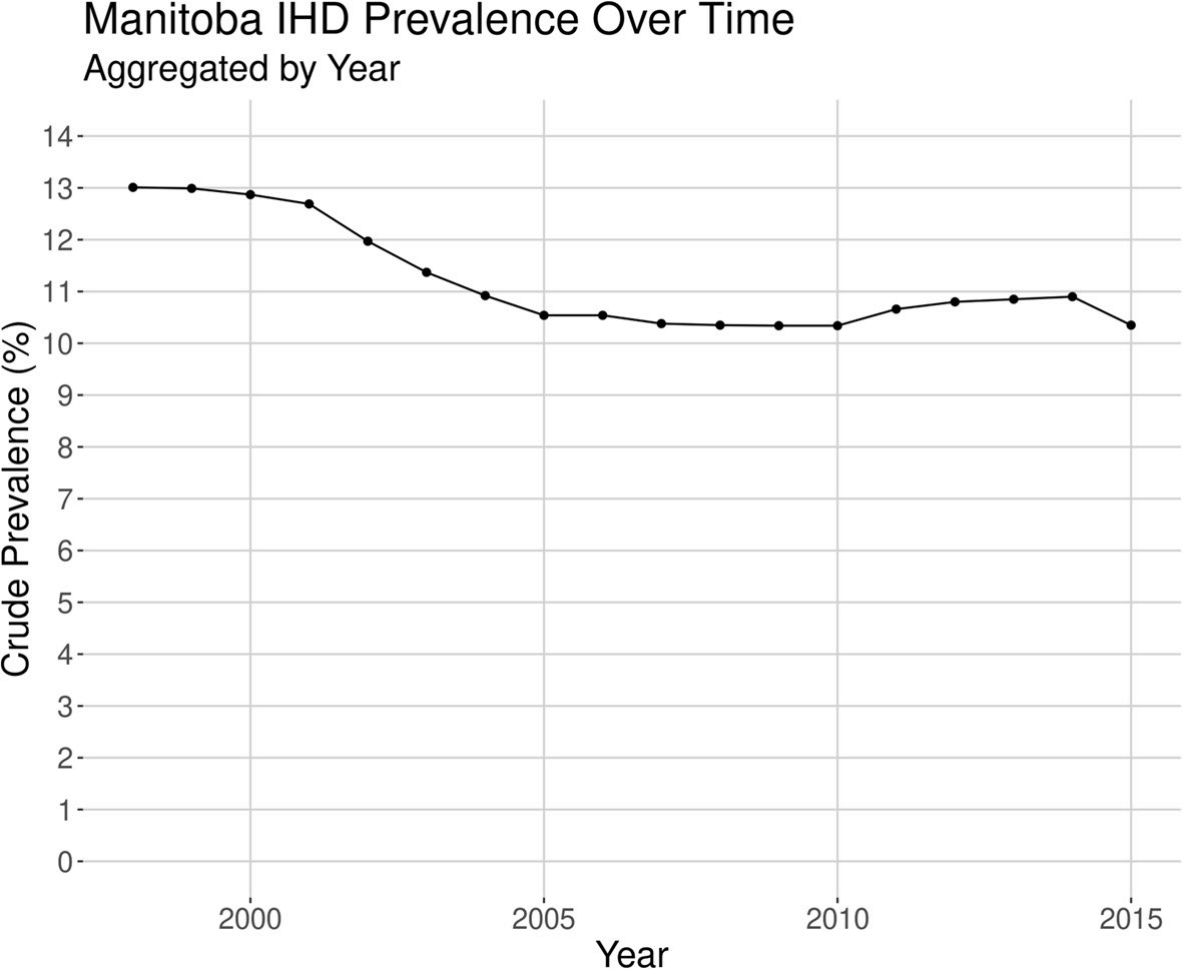
Crude IHD prevalence at the Provincial level, over the study period

The overall temporal trend for crude IHD prevalence during the study period was on average a decreasing trend, where the maximum occurred in the first year of the study (1998) with 13.01% prevalence, and the minimum occurred in 2010 with 10.35% prevalence, see Fig. 1 for the unadjusted prevalence rate over time.

**Fig. 2:**
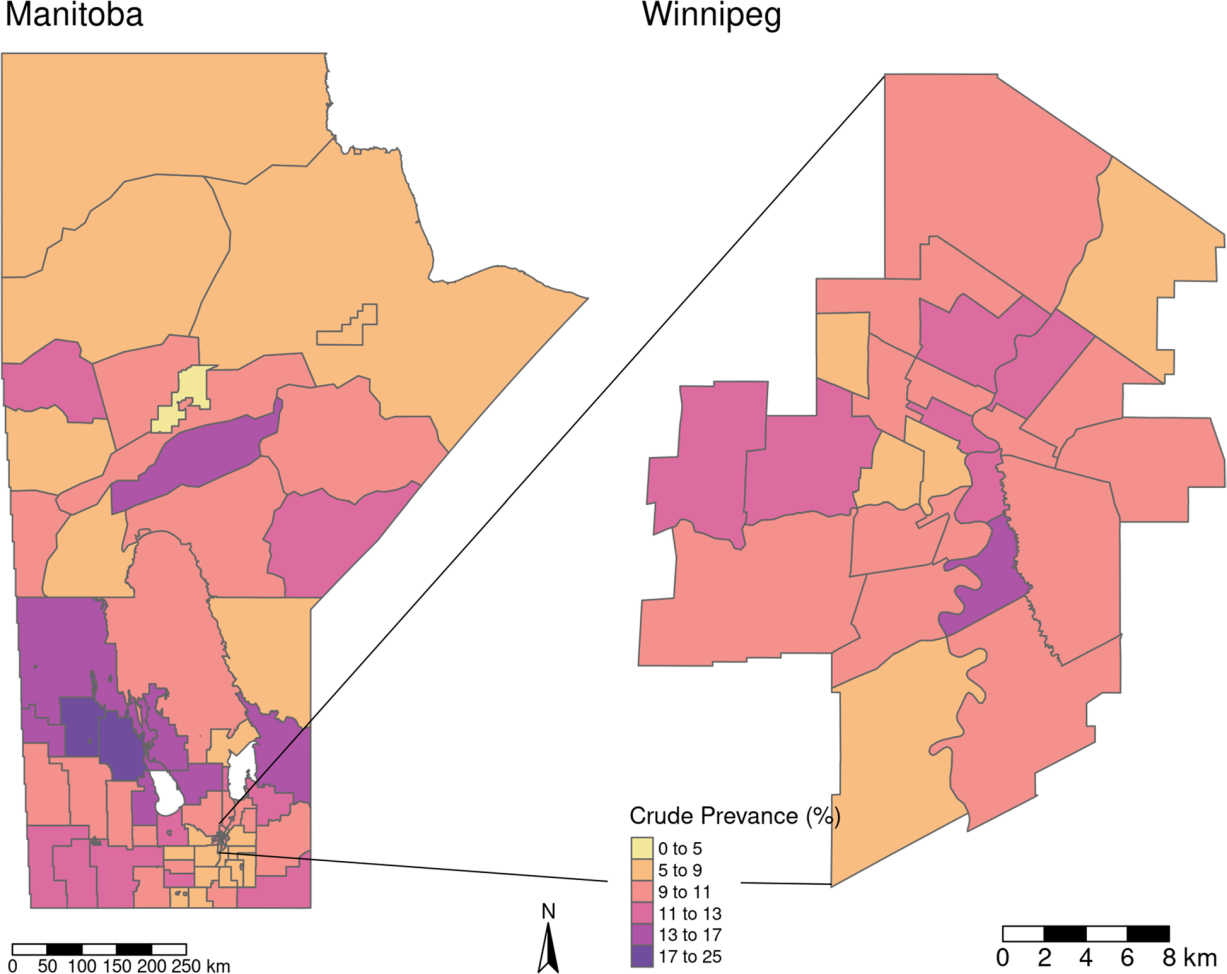
Crude prevalence rates at the RHAD level, for Manitoba in 2015

At the RHAD level, the maximum crude prevalence occurred in 1998 in the E Rural East RHAD; part of the Southern Regional Health Authority (RHA); with a prevalence of 24.88%, and the minimum occurred in 2008 in the W Stanley RHAD; also part of the Southern RHA; with a prevalence of 3.98%. Hence, disaggregating into smaller regions has shown large intra-RHA variance, which would have been averaged out if taken at the RHA level. Figure 2 shows this variation in the crude prevalence rates for Manitoba in 2015.

## Social Determinants and Spatial Variation of IHD

**Fig. 3:**
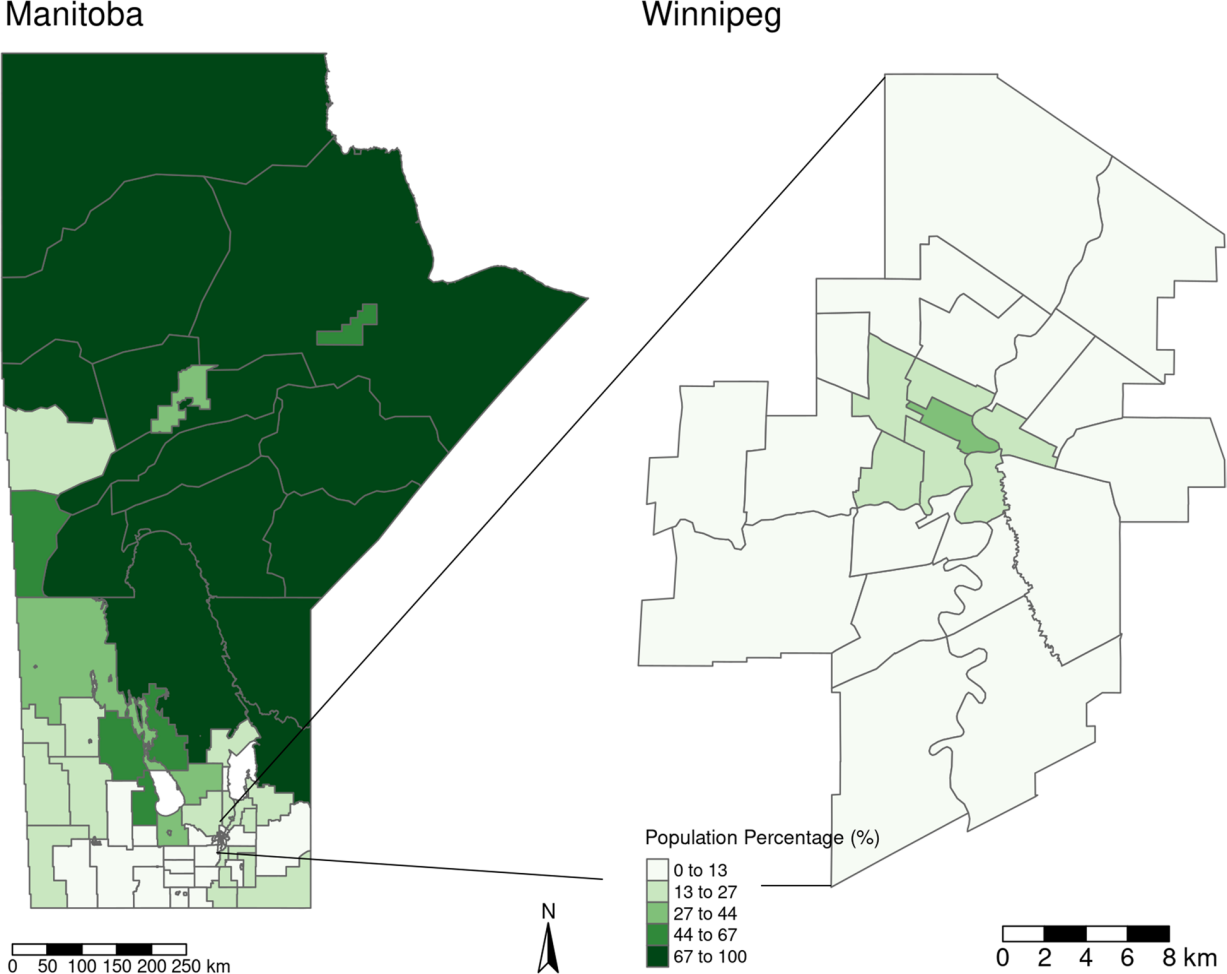
Indigenous population proportion for each RHAD, as calculated from the 2016 Canadian Census

**Fig. 4:**
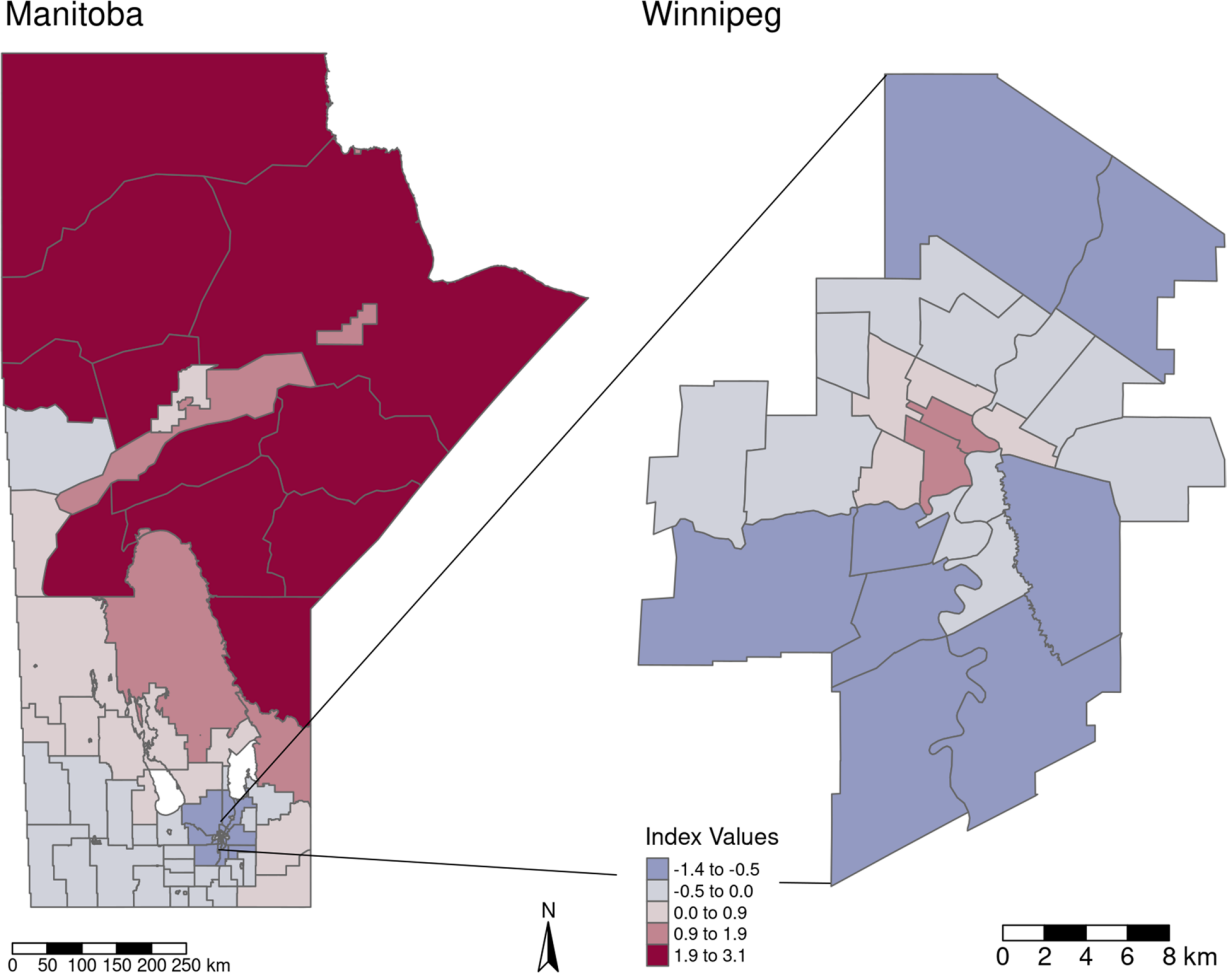
SEFI-2 mapped to RHAD’s. Calculated from the 2016 Canadian Census, this index is the scores from a factor analysis of 4 census variables: Proportion of lone parent families, median household income, unemployment rate, and proportion of adults without a high school diploma. Larger values indicate greater socio-economic deprivation

Regional population proportions of Indigenous populations were much higher in northern and north-eastern Manitoba and lower in the Southern part of the Province. SEFI-2 differed between the northern regions of Manitoba and central Winnipeg compared with the remainder of the province, with higher values of the index corresponding to greater socioeconomic deprivation in these regions. Many regions with higher proportions of Indigenous populations also had higher levels of socio-economic deprivation, with correlation between these two measures at the RHAD level being 0.92 (p-value < 0.001). Figures 3 and 4 display the geographical distribution of Indigenous population proportion and SEFI-2 variables respectively.

**Fig. 5:**
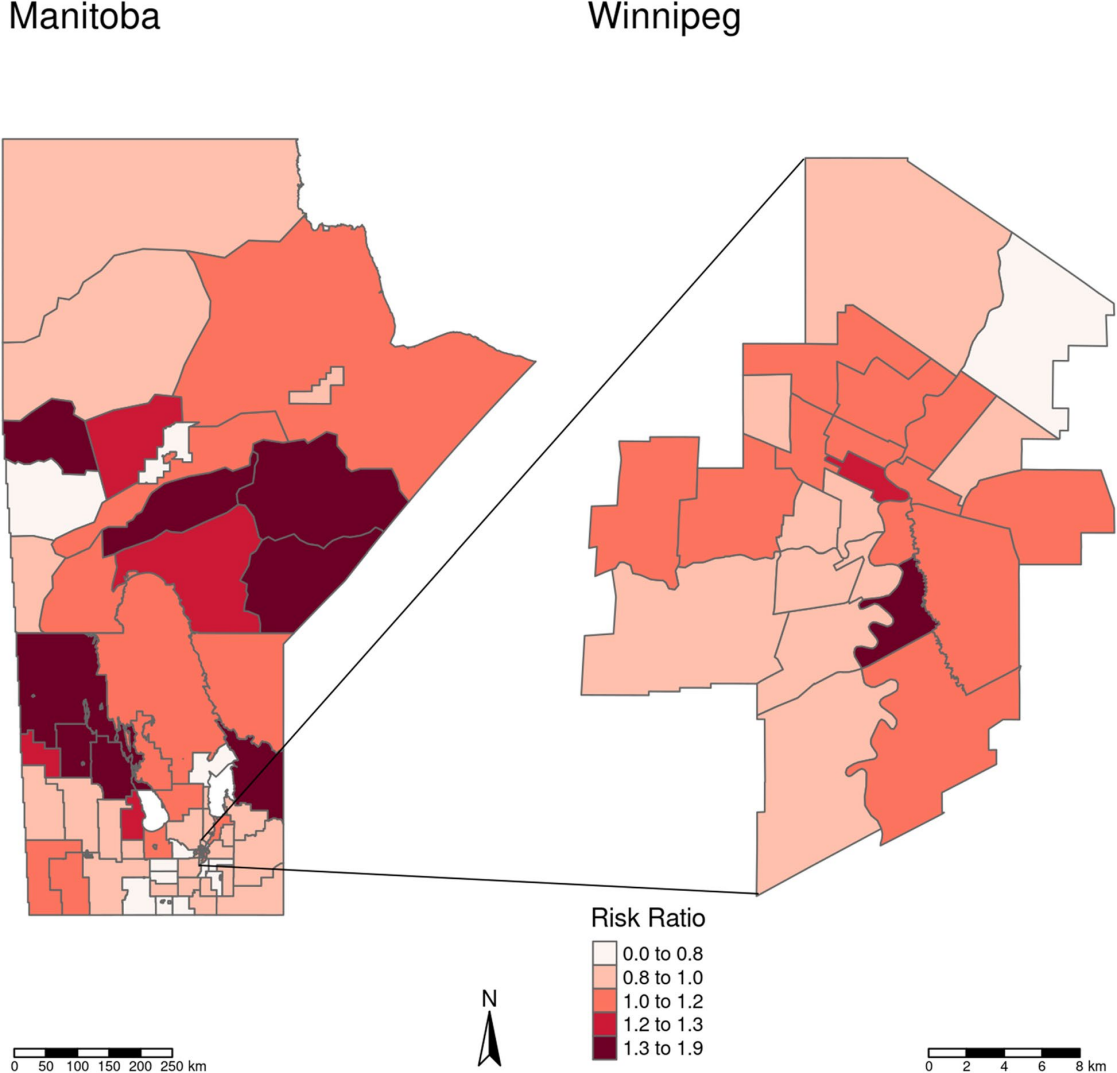
Model fitted age- sex-standardized risk ratio’s of IHD prevalence in Manitoba during the year 2015, calculated by the univariable model with Indigenous population proportion as the covariate

**Fig. 6:**
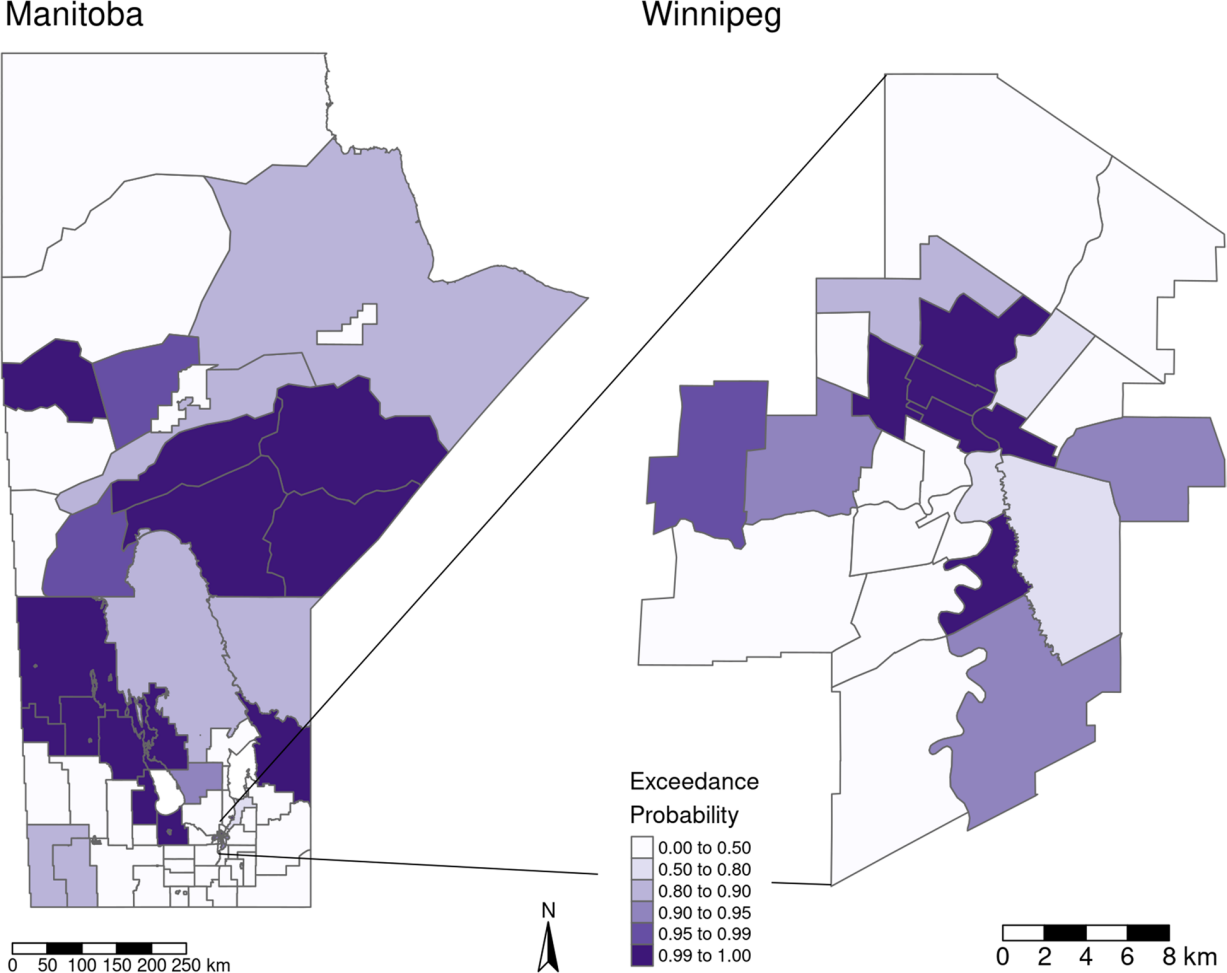
Exceedance probabilities for the univariable model with Indigneous population proportion as the covariate. Probabilities represent the probability that the age- sex-standardized risk ratio of IHD prevalence in Manitoba during the year 2015 in a given region is above 1 (null), or Pr(RR >1)

**Fig. 7:**
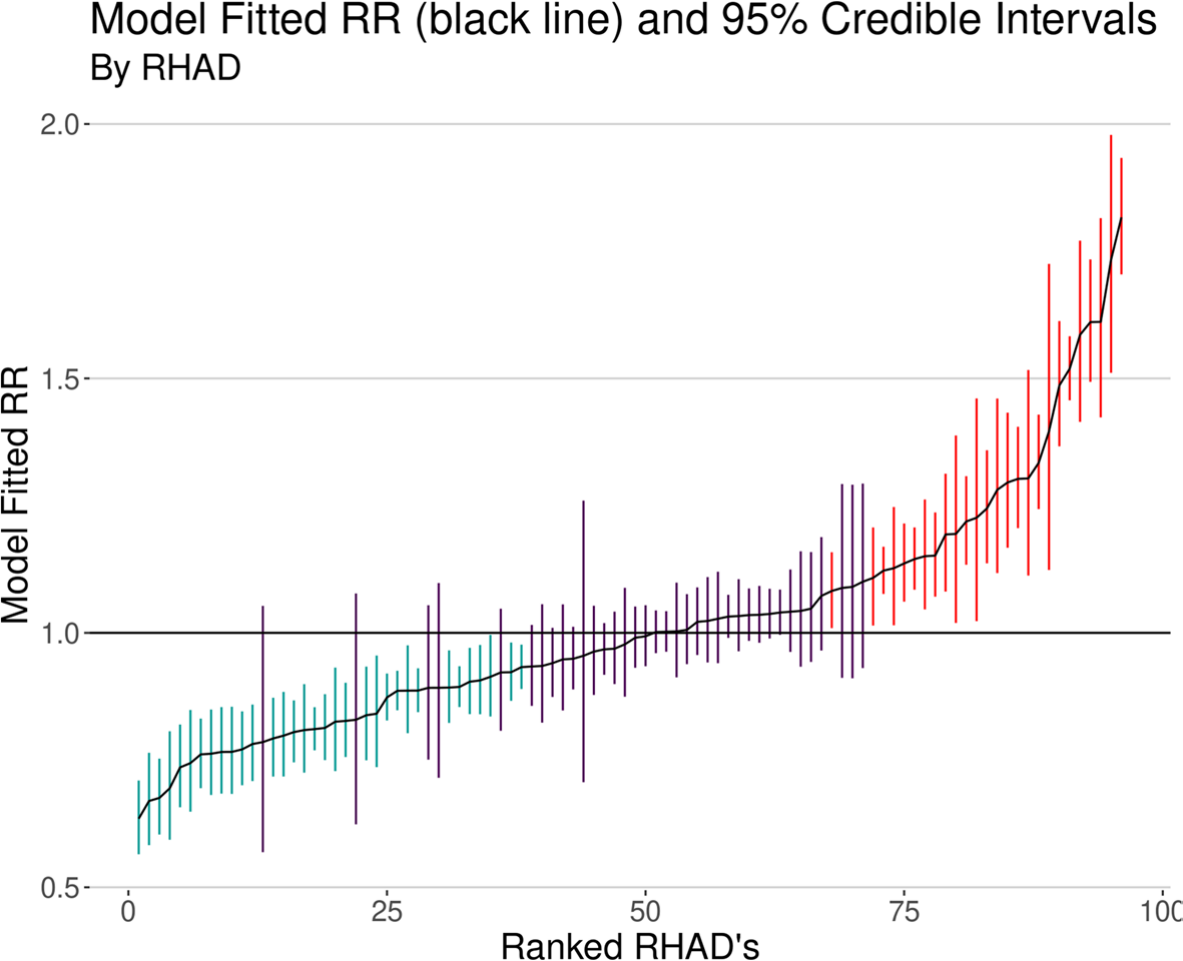
95% credible intervals for the age-sex standardized RR’s for each RHAD, ranked by the fitted median RR for each RHAD. Blue bars indicate RHADs with significantly lower RR than the Provincial average, red indicates significantly higher risk ratios and purple indicates RHADs with no significant difference from the Provincial average

Table 1 gives the model outputs of each of the univariable models as well as the multivariable model given by Eq. (1). Both covariates, Indigenous population proportion and SEFI-2, were significantly associated with increased IHD Risk Ratio (RR) in the univariable models with increased Indigenous population proportion and increased socio-economic deprivation associated with increased IHD prevalence risk. However, SEFI-2 was not significantly associated with IHD prevalence in the multivariable model, where the 95% credible interval covers a RR of 1. Judging by the DIC (lower is better) and CPO (higher is better) the univariable model with Indigenous population proportion marginally outperformed the other models, indicating that the SEFI-2 variable wasn’t adding much to the performance of model fit. There was a small detectable amount of confounding between the Indigenous population proportion and SEFI-2 variables, as the shift in the Indigenous population proportion coefficient estimate was approximately 2% from the univariable to the multivariable model. For the best performing model, the univariable model with Indigenous population proportion as the covariate, 98.5% of the model variance was attributed to the spatially structured random effects term and 1.5% to the unstructured term. This indicates a large degree of spatial confounding is accounted for in the model, and also that very little of the spatial variance is accounted for by the covariates. Figure 5 maps the fitted univariable model with Indigenous population proportion as the covariate (best performing model), and Fig. 6 maps the exceedance probabilities of this model, where there are visible clusters of regions with high probabilities of having elevated prevalence rates of IHD. Exceedance probabilities are defined as the probability that the age- sex-standardized risk ratio of IHD prevalence in a given region is above 1, or Pr(RR >1). Figure 7 also shows the credible intervals for each RHAD’s fitted risk ratio.

**Table 1:** Spatial model outputs. RR of SEFI-2 corresponds to an increase of 1 in the index measure. RR of Indigenous population proportion corresponds to an increase of 0.1 in population proportion

**Covariate**	**RR (95% Credible Interval)**		**DIC**	**CPO**
**Univariable Models**				
Indigenous Population Proportion	1.07	(1.05, 1.11)	935.27	-527.95
SEFI-2	1.19	(1.12, 1.26)	937.18	-531.75
**Multivariable Model**				
Indigenous Population Proportion	1.05	(1.02, 1.09)	935.63	-528.12
SEFI-2	1.07	(0.99, 1.13)		

## Temporal and Spatio-Temporal Patterns

Table 2 displays the percentage of variance from the random effects terms as well as the model fit criterion from the two spatio-temporal models given by model Eqs. (2) and (3). The model without the space-time interaction term had 96.6% of its variation attributed to the structured spatial term, and only 0.07% to the structured temporal term, indicating the lack of a significant overall time trend in IHD prevalence

**Table 2:** Spatio-Temporal model assessment criteria; percentage (%) of variance explained by each random effects term, DIC, and CPO

Random Effects Term	Model with no Interaction	Model with Interaction
Unstructured Spatial	3.29	26.37
Structured Spatial	96.60	26.22
Unstructured Temporal	0.04	0.45
Structured Temporal	0.07	1.30
Space-Time Interaction	-	45.66
**Model Fit Parameter**		
DIC	18549.42	14919.04
CPO	-9399.89	-7370.87

Judging by both the DIC and CPO, the model with the space-time interaction term performed much better at fitting the data. This interaction term also soaked up 45.66% of the model variance, indicating that there were significant differences in temporal trends over the areal units. The structured temporal random effects term still had a minimal share of the model variance, indicating that little of the model variance was explained by the overall temporal trend even after accounting for differences in time trends between areal units.

**Fig. 8:**
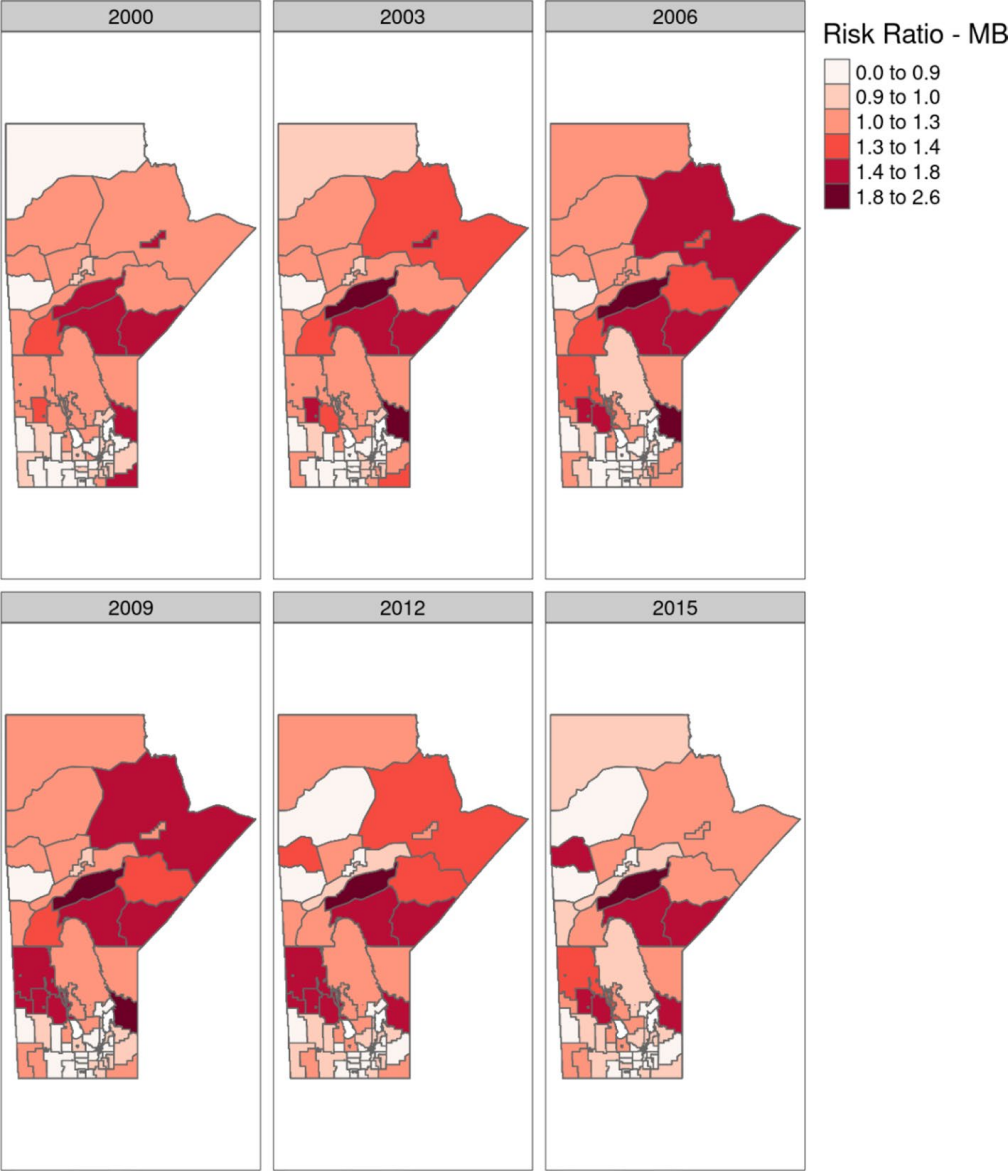
Age-sex adjusted RRs for Manitoba, fitted by the spatio-temporal model with the space-time interaction term. Six evenly spaced time points are displayed for ease in map readability

**Fig. 9:**
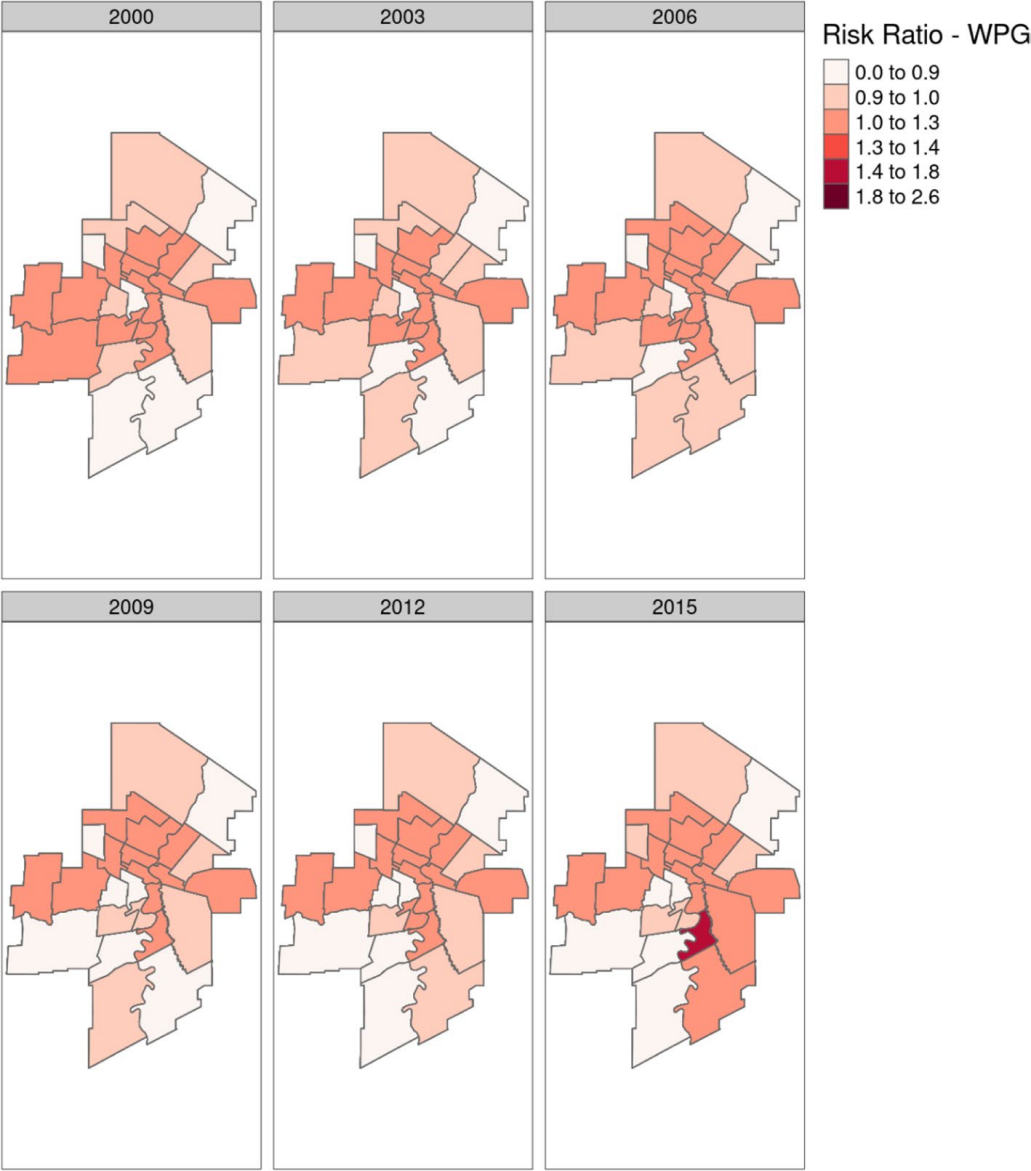
Age-sex adjusted RRs for Winnipeg, fitted by the spatio-temporal model with the space-time interaction term. Six evenly spaced time points are displayed for ease in map readability

**Fig. 10:**
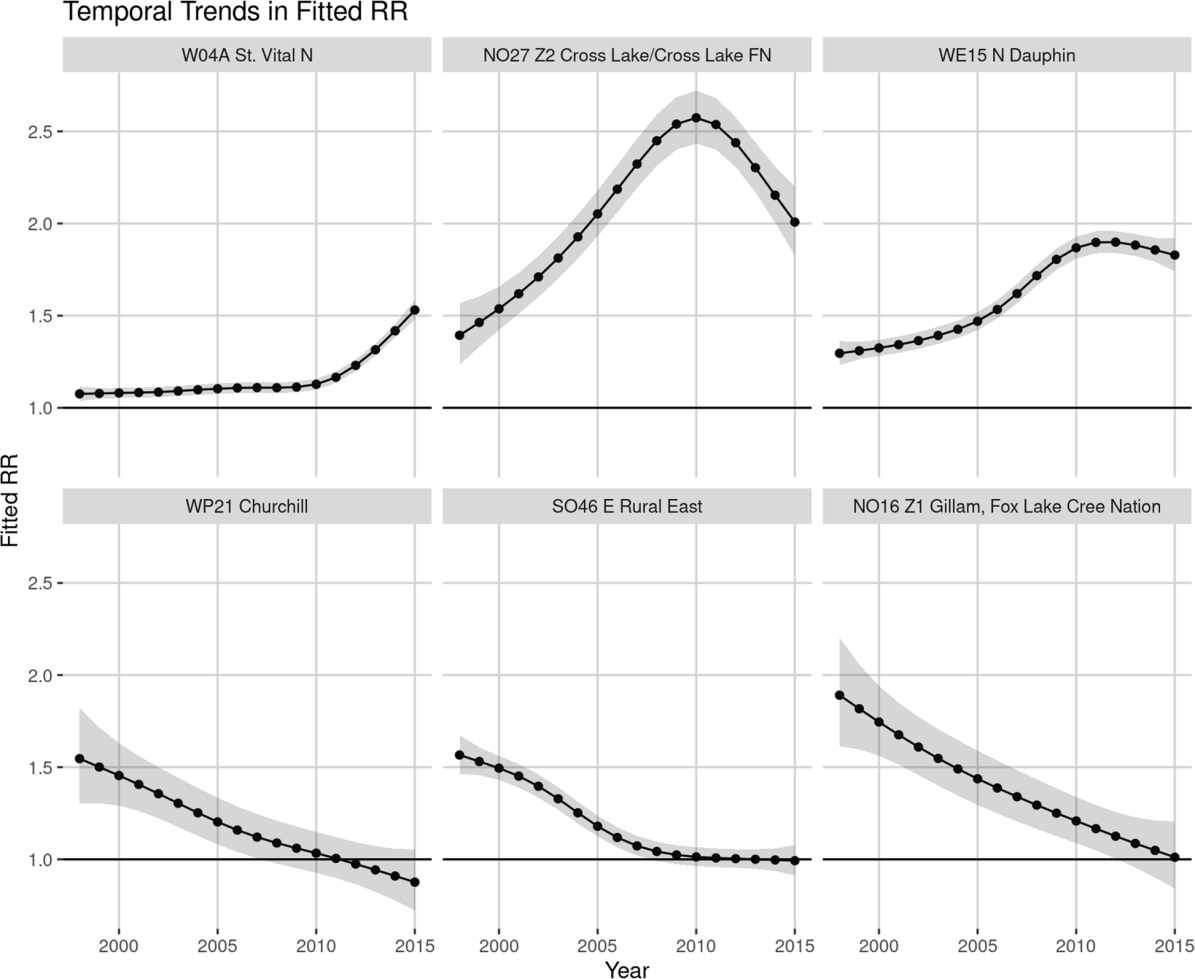
Fitted temporal trends for selected regions. Top 3 plots represent regions that had the largest increase in risk over the time period, and the bottom 3 plots represent regions that had the largest decrease in risk over the time period

Model output for the model with the space-time interaction term is included in Figs. 8 and 9. Here six evenly spaced time points from the model are shown, for both Manitoba as a whole and Winnipeg individually. This shows regions with trending RR’s when compared with the Provincial average at each time point. Figure 10 shows three RHADs that had the largest increasing trend and the three RHADs that had largest decreasing trends, indicating significant differences in the temporal trends between regions.

## Discussion

In this study, which was a continuation of the M.Sc. thesis by Dyck entitled Spatial Analysis of Ischemic Heart Disease in Manitoba [27], we have shown that higher proportions of Indigenous populations and lower SES, as measured by SEFI-2, both individually contribute to higher risk of IHD prevalence within the 96 RHAD’s in Manitoba when accounting for the confounding effect of the spatial covariance structure of the data. When modelled together, SEFI-2 was not significant indicating it wasn’t explaining any extra variation in IHD prevalence that the Indigenous population covariate wasn’t already explaining. Visually comparing the results in Figs. 5 and 6 with figure, it can be seen that the patterns in IHD risk occur predominantly in RHAD’s that have high Indigenous population proportions. These findings align with the analysis of research by Tobe et al. (2015) on the risk of cardiovascular disease in Indigenous populations. Our findings show in Table 1 that even when controlling for spatial confounding and SES with the SEFI-2 variable, regions with higher Indigenous population proportions had significantly higher risk of IHD prevalence. .

There was visual evidence of a decreasing Provincial trend for crude IHD prevalence (Fig. 1), especially at the beginning of the study time period (1998-2005), however the spatio-temporal models did not confirm this, as there was no evidence of a global trend in the spatio-temporal model when accounting for space-time interactions. An attempt was made to model the first eight years of the data (1998-2005). However, as before, no global trend was detected after accounting for the space-time interaction effect. This finding is contrary to “The 2013 RHA Indicators Atlas” which showed a significant decrease in IHD prevalence from the 2002-2007 to 2007-2012 time periods, where the Provincial prevalence significantly decreased from 8.8 to 7.9% over this time period [1]. However, our study models the data over time and space, accounting for covariance between regions as well as between time points, adding a higher level of robustness to our analysis.

Evidence of a strong spatial covariance structure was present in the data, with 95.6% of the model variance being accounted for by the spatially structured random effects term when excluding the space-time interaction random effects term. However, when modelling the data with space-time interaction term this attenuates to 26.22%, where 45.66% of the model variance is attributed to this space-time interaction term. This indicates that the spatial covariance structure significantly changes temporally over the study period, as well as the temporal covariance structures significantly varying between regions.

Temporal trends were significant at the regional level, and varied significantly between regions. This is most likely the reason behind our previous conclusion, that no Provincial trend was detectable, as some regions had significant increases and some significant decreases in IHD prevalence over the study period. Figures 8 and 9 showed this space-time distribution, where clusters of disease appeared in the Western part of the Province during the mid to later part of the study period, and then tapered off towards the end. Time-series plots of the model fitted IHD RR’s for regions that had significant trends are given in Fig. 10. As is seen in this figure, there are large differences in temporal patterns between these regions. Full model output for each region is provided in the supplemental materials for readers interested in specific regions.

A strength of this study was the ability of the models to capture the spatial, temporal, and space-time interaction covariances. These models produce more accurate measures of association between the covariates and the response as well, as these measures of association are usually inflated in prediction magnitude when model assumptions are not met in the conventional Poisson regression model context.

Another strength was the quality of the data from the PRDR, as this captures an accurate snapshot of IHD prevalence over time at the population level. As this is a Province wide administrative data system, most health care system interactions within the Province are captured in this repository, providing a high level of accuracy for IHD diagnoses within the Province. This allowed for precise spatial and temporal assignment of persons who previously had an IHD diagnosis. Breaking this into yearly segments allowed us to identify temporal patterns of IHD prevalence within the individual regions.

A main limitation of this study is the usage of aggregated data to model IHD prevalence. Here, the underlying continuous process of disease occurrences is discretized into 96 aggregated areal units. By using aggregated data this project is an ecological study and there is the risk of ecological fallacy. As this is still an ecological study, all conclusions are made at the regional level and one cannot infer about the individual risk levels for persons living within a region. Another minor limitation is the changes in regional borders over time. A small part of the population will have been re-classified due to changes in Postal Code boundaries that make up each RHAD. However, these differences are minor, where only a small handful of Postal Codes in rural areas were affected over the study period.

The quality of the administrative data should be considered in the context of research projects, as by their very nature are meant for a different purpose from that of health services or public health research. The trend in recent time has been to increase the number of shadow-billed physicians, contrasted to fee-for-service physicians, in northern Manitoba. In Manitoba, shadow billing occurs when salary paid Physicians submit records of services completed, whereas fee-for-service paid Physicians receive payment for each service delivered and submitted to the Province for payment. This may bias the number of true IHD cases, as it has been shown that shadow-billed physicians are less reliable in their reporting [28]. It was shown in Alshammari & Hux (2009) that diabetes reporting by shadow-billed physicians was biased towards extreme cases; less-extreme cases were under-represented compared to their fee-for-service counterparts. This under-reporting could result in biasing disease rates in aggregated regions towards the mean. Some of this effect would be partly mitigated with the “information borrowing” of the neighbourhood structures used in the MPM, but it is noteworthy to mention as this could effect the outcome of the measured rates in some of the northern regions.

## Conclusions

The results of this study will be useful for health system policy makers and planners when making decisions about health care delivery. The identification of regions with elevated IHD prevalence can provide a better representation of where IHD is affecting Manitobans with greater magnitude than the rest of the province. Identifying regions whose temporal trends were counter to the visually observed provincial decreasing trend of IHD prevalence, could also aid in evaluating where certain policies that were designed to decrease heart disease are working and where more work is needed.

Another of Manitoba Health, Seniors & Active Living’s priorities is to “Lead advances in health service delivery with First Nations, Métis, and Inuit Manitobans, through policy and programs with a focus on prevention, primary health care, public health, and education” [29]. As the spatial models for the 2015 data showed in Table 1 that regions with larger Indigenous population proportions had higher risk of having elevated IHD prevalence, this study points towards a health outcome where disparities between Indigenous populations and the rest of Manitobans exist, even after accounting for SES indicators and other latent spatially correlated environmental factors. Thus, to meet this priority, a reduction in IHD among the Indigenous population in Manitoba is necessary. This study provides information on which regions policy could be focused on and prioritized to achieve the greatest impact.

## Supplementary Information

**Additional file 1.** Supplementary Material 1.

**Additional file 2.** Supplementary Material 2

**Additional file 3.** Supplementary Material 3.

**Additional file 4.** Supplementary Material 4.

**Additional file 5.** Supplementary Material 5.

**Additional file 6.** Supplementary Material 6.

**Additional file 7.** Supplementary Material 7.

**Additional file 8.** Supplementary Material 8.

**Additional file 9.** Supplementary Material 9.

## Data Availability

The data that support the findings of this study are available from the Manitoba Centre for Health Policy but restrictions apply to the availability of these data, which were used under license for the current study, and so are not publicly available. Data are however available from the authors upon reasonable request and with permission of the Manitoba Centre for Health Policy.

## Abbreviations

BYM: Besag-York-Mollie [model]
CPO: Conditional Probability Ordinate
DIC: Deviance Information Criterion
DPIN: Drug Program Information Network
MPM: Mixed Poisson Model
GMRF: Gaussian Markov Random Field
IHD: Ischemic Heart Disease
INLA: Integrated Nested Laplace Approximation
MCHP: Manitoba Centre for Health Policy
MCMC: Markov Chain Monte Carlo
PHIN: Personal Health Identification Number
PIT: Predictive Integral Transform
PRDR: Population Research Data Repository
RHAD: Regional Health Authority District
RR: Risk Ratio
SEFI-2: [second version of the] Socio-Economic Factor Index
SES: Socio-Economic Status
SRR: Standardized Risk Ratio
