# Supplementary figures and images for: Social determinants and spatio-temporal variation of Ischemic Heart Disease in Manitoba
Source: BMC Public Health. 2021 Dec 30;21:2325. doi: 10.1186/s12889-021-12369-1 (PMC8717667; doi:10.1186/s12889-021-12369-1)

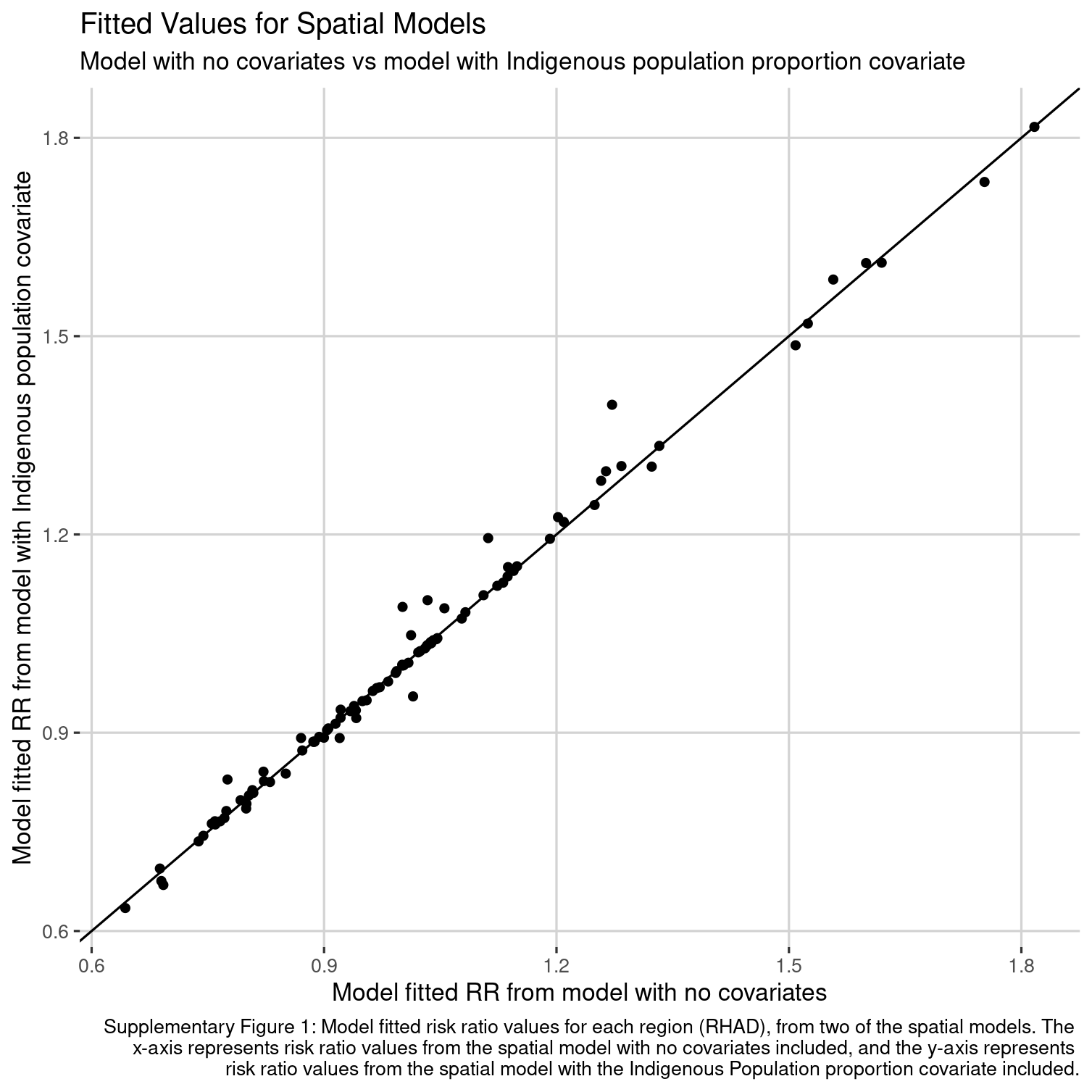

Supplement: Supplementary file 1 — Additional file 1. Supplementary Material 1. [file 12889_2021_12369_MOESM1_ESM.tiff]

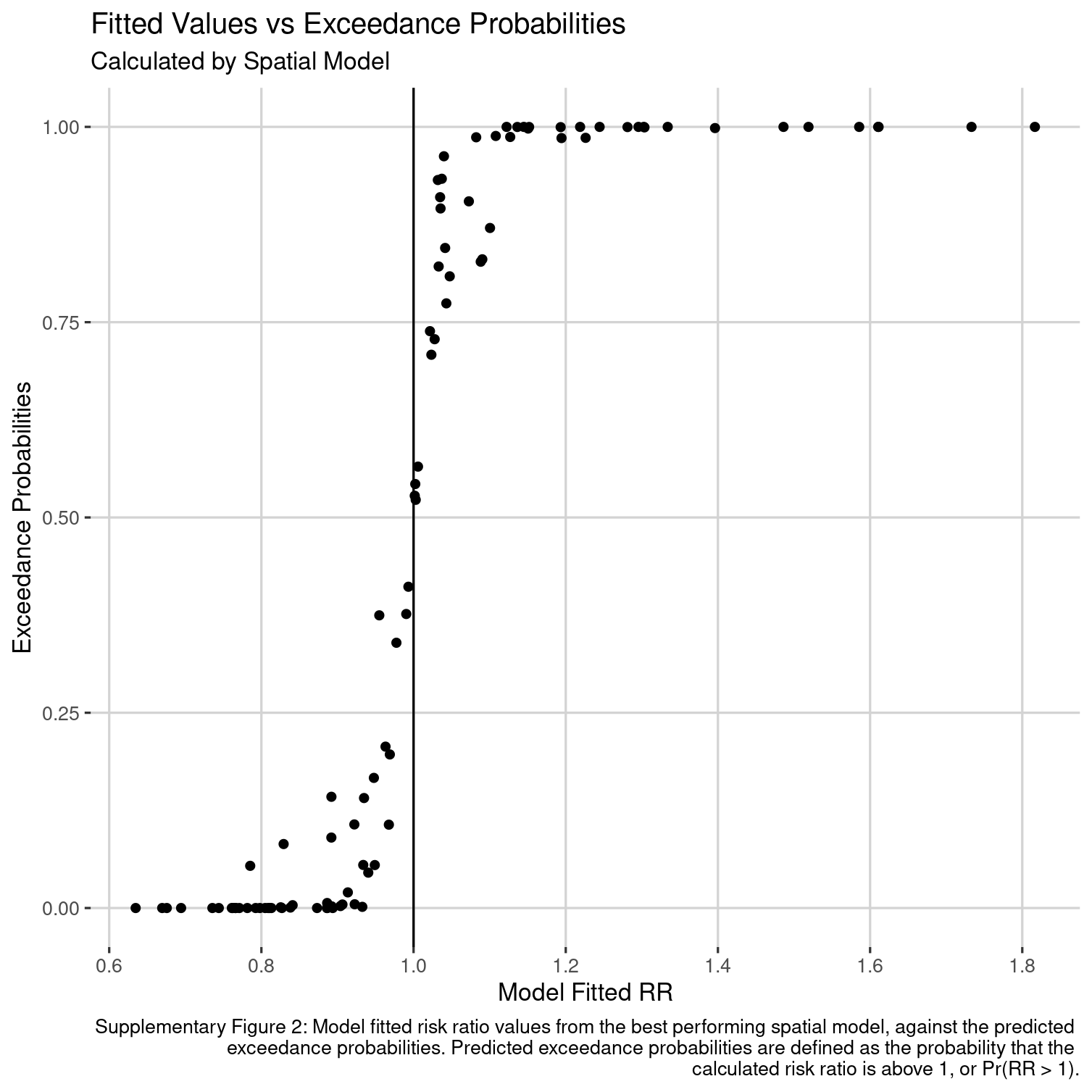

Supplement: Supplementary file 2 — Additional file 2. Supplementary Material 2 [file 12889_2021_12369_MOESM2_ESM.tiff]

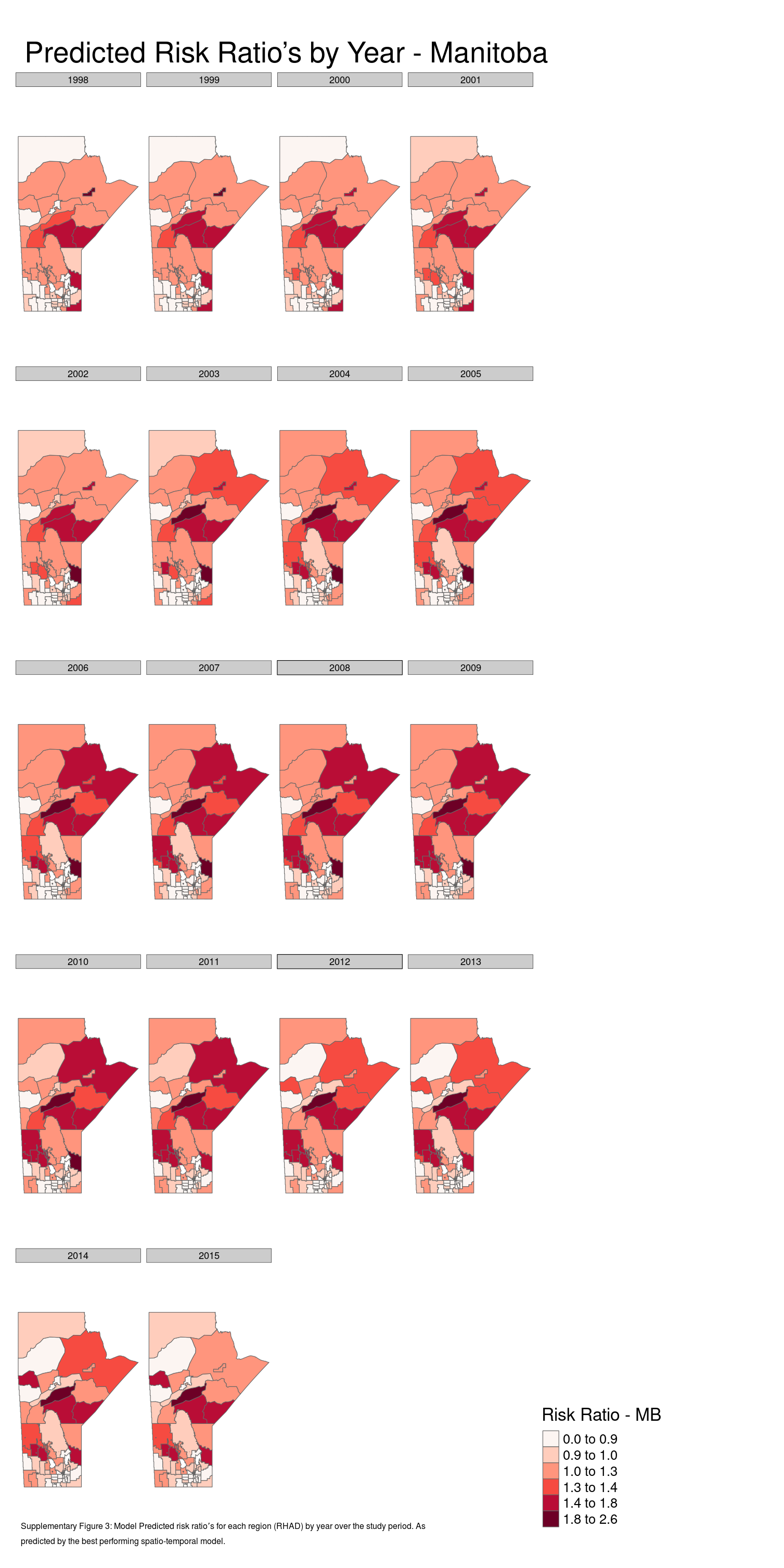

Supplement: Supplementary file 3 — Additional file 3. Supplementary Material 3. [file 12889_2021_12369_MOESM3_ESM.tiff]

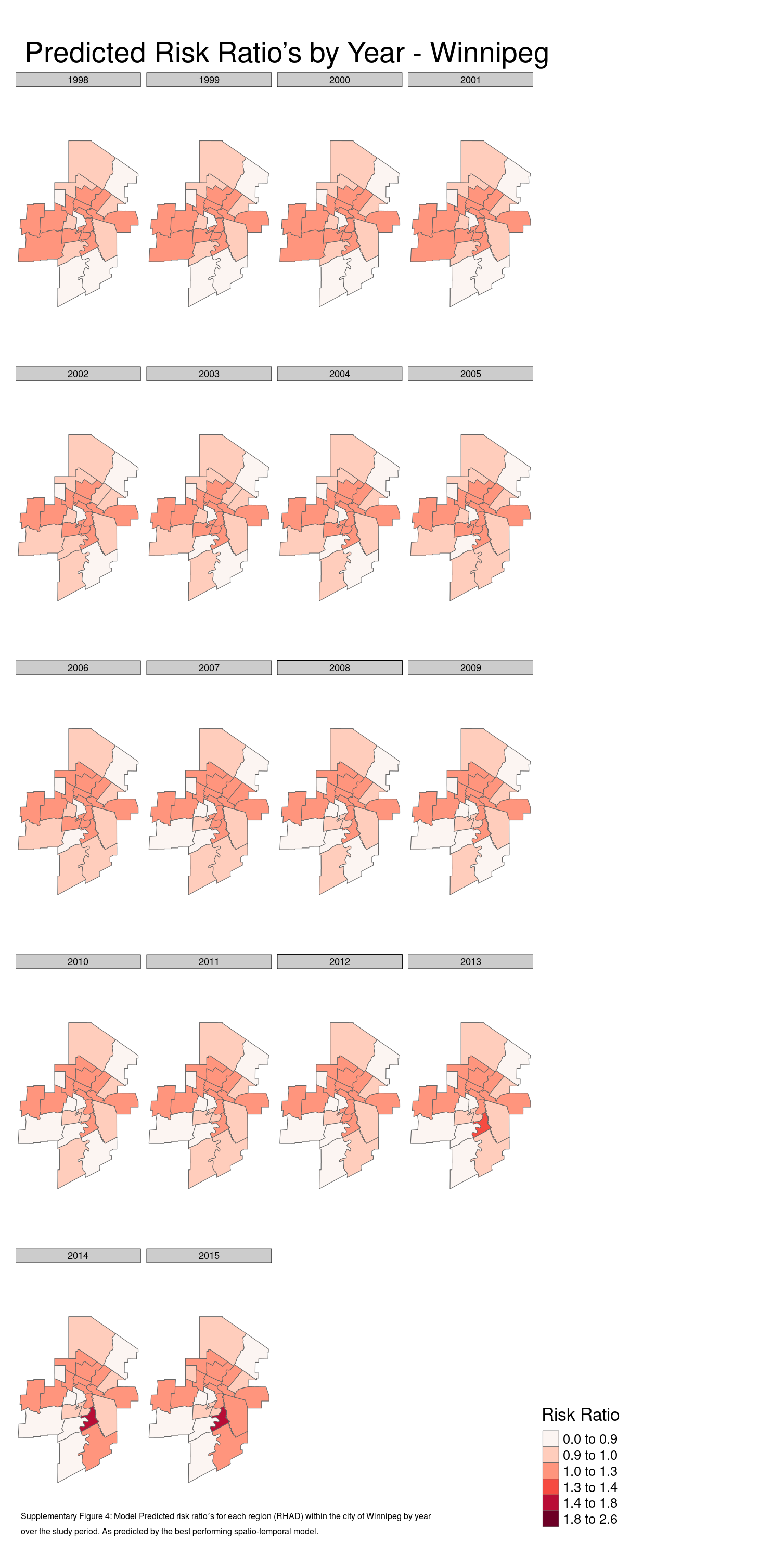

Supplement: Supplementary file 4 — Additional file 4. Supplementary Material 4. [file 12889_2021_12369_MOESM4_ESM.tiff]

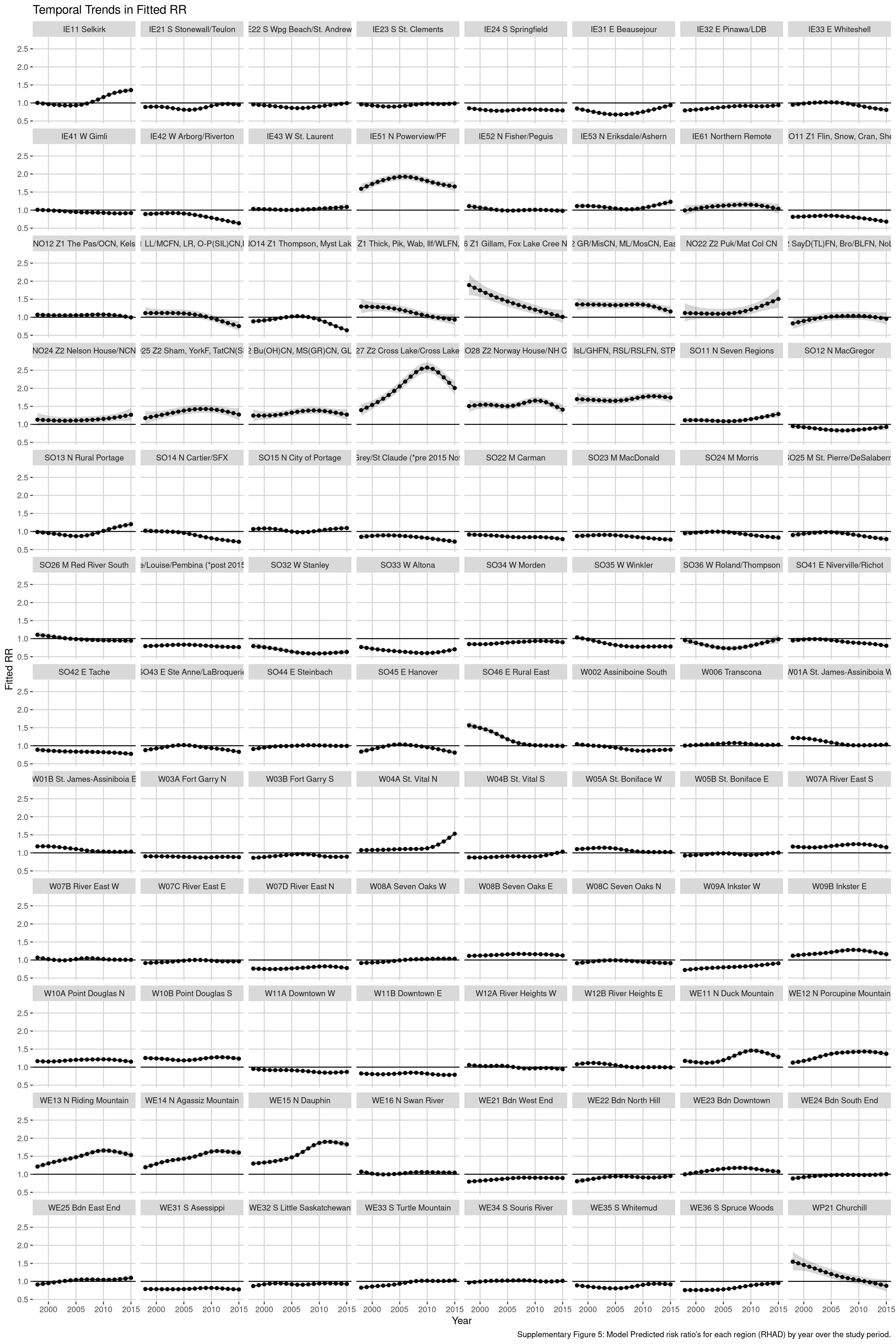

Supplement: Supplementary file 5 — Additional file 5. Supplementary Material 5. [file 12889_2021_12369_MOESM5_ESM.tiff]
